# Precision and reproducibility of blood T1 estimation: implications of T1 star on ECV calculation

**DOI:** 10.1186/1532-429X-17-S1-P4

**Published:** 2015-02-03

**Authors:** Anish N Bhuva, Thomas A Treibel, Heerajnarain Bulluck, Joanne Simpson, Charlotte Manisty, James Moon

**Affiliations:** 1 The Heart Hospital Imaging Centre, London, UK

## Background

Extracellular volume fraction (ECV) calculation requires myocardial and blood T1 measurements to be acquired pre- and post-contrast administration. T1 star (T1*) may offer a more accurate estimation of blood pool T1 as it avoids look-locker correction, and so is theoretically a more robust measure of blood T1 where fresh spins are flowing into the image plane. Reproducibility of blood T1 and T1* has not been as extensively explored as myocardial T1. We compared the precision and reproducibility of blood T1* using a Modified Look-Locker Inversion recovery sequence (MOLLI), against standard T1 measurements.

## Methods

Twenty healthy volunteers (mean age 35+/-8 years, 7 females) were scanned on two separate occasions on the same day at 1.5T (Siemens Avanto). Native T1 maps were acquired in the four chamber (4Ch) and mid ventricular short axis (SAX) using a MOLLI 5s(3s)3s sequence, providing both T1 and T1* maps in the same acquisition. Regions of interest (ROI) were drawn separately by two investigators in the LV blood pool of the T1 map and copied onto the T1* maps. The variability of each sequence was expressed as an absolute percentage difference between both scans, divided by the mean. The coefficient of variability (CV) was calculated as the standard deviation of the difference divided by the mean of the parameter under consideration.

## Results

Blood T1* values were significantly lower than T1 values in 4Ch (1559±71ms vs 1610±64ms, p=0.0002), whereas they were significantly higher than T1 in SAX(1636±87ms vs 1613±64ms, p= 0.02) (Table 1).

**Table 1:** Precision and correlation of T1* compared to T1 blood pool

	Mean ± SD/ms			
	T1	T1*	p	R
4 chamber SAX	1610±64 1613±64	1559±71 1636±87	0.0002 0.02	0.80 0.81

p R	NS 0.86	<0.0001 0.76		

**Figure 1:**
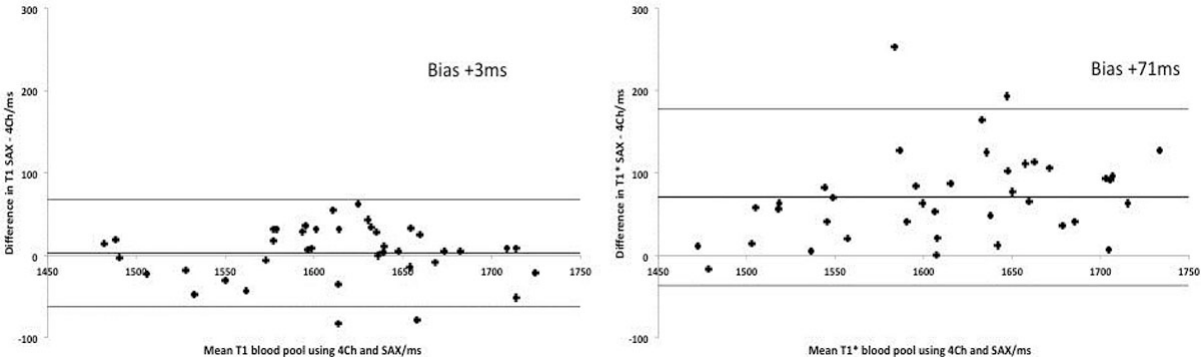
Bland Altman graphs showing increased variability and bias between views for T1S with little bias or variability for T1 of blood pool

There was no difference in MOLLI T1 between 4Ch and SAX blood pool with excellent correlation (p=0.54, R 0.86) and little bias through Bland-Altman analysis. MOLLI T1* had significantly higher SAX than 4Ch blood pool values with good correlation (p<0.0001, R 0.76) (Figure 1).

There was significantly less inter-scan variability in blood T1 and T1* measurements in SAX (~0.02%) than 4Ch (~1.6%) views (p<0.0001 for both). MOLLI T1 showed significantly less inter-scan variability than T1* in SAX measurements (p=0.001, CV 4% vs 6%), but not in 4Ch measurements (p=0.5, CV 4% vs 4%).

There was excellent inter-observer reproducibility for both SAX T1 and T1* (Intraclass correlation coefficient (ICC) 0.994, 95% CI 0.984-0.998 vs ICC 0.978 95% CI 0.94-0.991). 4Ch T1 however was slightly more reproducible than T1* (ICC 0.981, 95% CI 0.953-0.992 vs 0.964, 95% CI 0.909-0.986).

## Conclusions

For ECV calculation, T1 and T1* cannot be used interchangeably. Whilst T1* star is theoretically more accurate, MOLLI T1 is more reproducible, most notably when there is in plane rather than through plane flow. For both sequences, short axis blood pool measurements were more precise and reproducible than four chamber, and therefore this should be adopted as the standard view for T1 blood measurement.

## Funding

N/A.

